# Prevalence and Management of Septic Shock among Children Admitted at the Kenyatta National Hospital, Longitudinal Survey

**DOI:** 10.1155/2019/1502963

**Published:** 2019-12-17

**Authors:** Varsha Vekaria-Hirani, Rashmi Kumar, Rachel N. Musoke, Ezekiel M. Wafula, Idris N. Chipkophe

**Affiliations:** ^1^Department of Paediatrics and Child Health, University of Nairobi, P.O. Box 19676-00202, Nairobi, Kenya; ^2^Department of Paediatrics and Anesthesia, Kenyatta National Hospital, P.O. Box 20723-00202, Nairobi, Kenya

## Abstract

**Background:**

Paediatric septic shock is a subset of sepsis associated with high mortality. Implementing the existing international Surviving Sepsis Campaign Guidelines 2012 (SSCG) have contributed to reduction of mortality in many places but these have not been adopted in our setting. The current study aimed at documenting the practice at a national referral hospital.

**Methods:**

A hospital based longitudinal survey carried out among 325 children from September to October 2016. Children aged 0 days (≥37 weeks gestation) to12 years were included. The aim was to determine the prevalence, audit the management and determine the outcome at 72 hours of septic shock among children admitted at the Kenyatta National Hospital (KNH). A standard questionnaire was used for data collection and Surviving Sepsis Guideline 2012 was used as a reference for auditing the management of septic shock. Data was stored in MS-EXCEL and analysed in STATA 12.

**Results:**

The prevalence of septic shock was 50 (15.4%), with a median age of 4 months. Septic shock was recognized by the attending clinician in 28 (56%). The level of care to children with septic shock was not to the level recommended by the SSCG 2012. Odds of being diagnosed with septic shock reduced with age (odds ratio 4.38 (1.7–11.0), *p* = 0.002) and no child aged above 60 months age was diagnosed with septic shock. The mortality was 35 (70%) at 72 hours of admission, with a median of 14 hours. Infants had the highest case fatality of 82.6%. It was found that lack of mechanical ventilation, and presence of hypotension at admission were associated with greater mortality (*p* values of 0.03 and 0.01 respectively).

**Conclusion:**

The prevalence rate of septic shock is 15.4% among children admitted at the KNH and is associated with high mortality. The advanced degree of shock contributed to mortality. The level of care at KNH was not to the level of SSCG 2012, and hence the need to include septic shock management guidelines/protocols in our local Kenyan paediatric guideline.

## 1. Introduction

Paediatric septic shock, a subset of sepsis, is accompanied by cardiovascular and cellular dysfunction and is associated with high mortality globally [1]. Sepsis is a syndrome of life threatening organ dysfunction caused by dysregulated host response to infection. Clinical signs needed to recognize septic shock include signs of suspected sepsis, systemic inflammatory response syndrome and altered tissue perfusion [2, 3]. This is yet to be validated for definition of paediatric septic shock. Good knowledge and a high index of suspicion are required in early recognition of septic shock as the diagnosis may easily be missed or delayed [1–4].

Studies in multiple settings have shown varying figures for prevalence of septic shock among children admitted to paediatric/neonatal intensive care unit (PICU/NICU). Sepsis and septic shock affect millions of children every year globally and killing one in four [1]. Prevalence rates of 2.2% of all paediatric admissions and 18.4% of PICU admissions have been cited in studies from India [5, 6]. There is paucity of data on prevalence of paediatric septic shock in African countries and no studies have been done on prevalence of septic shock in children in the continent.

Early recognition of septic shock remains the key to reduction of mortality among children [1]. Han et al. reported a 9-fold improvement in survival when septic shock was reversed early while every additional hour of delay in shock reversal was associated with >2-fold odds of mortality [7]. Audit on early goal directed management have shown marked improvement in mortality after introduction of Surviving Sepsis Guidelines. A study done by Zambon et al. found that compliance to guidelines reduced mortality from 41% to 16% and reduced length of stay from 9 to 5 days [8]. A 3-year period study in Bangkok after implementation of Surviving Sepsis Guidelines showed reduction in mortality from 42% to 19% [9].

Mortality from septic shock remains high worldwide and is influenced by the time of recognition and initiation of goal directed management [1, 2, 10–12]. Mortality remains high in the initial 72 hours of onset of sepsis and septic shock partly due to the hyper inflammatory phase (cytokine storm) of the immune response [13]. Presence of low arterial systolic blood pressure and PH, presence of disseminated intravascular coagulation and extent of multi-organ failure have been associated with poor outcomes [14]. A study in India showed a 96 hours mortality of 70% [15].

There are challenges in resource limited settings where unavailability of PICU/NICU facilities and appropriate critical care training may hinder implementation of Surviving Sepsis Guidelines. A study on the status of septic shock outcomes has not been done locally [1, 16]. Inadequate recognition leads to missed or delayed diagnosis. A review of African hospitals showed that only 67% of the Surviving Sepsis guideline can be implemented in African hospitals and only 1.5% of low and middle-income African hospitals can fully implement Surviving Sepsis Guideline due to limited recourses such as drugs, equipment, and disposable material required [17].

In Kenya the magnitude of the problem is not known. The Surviving Sepsis Guidelines are used locally for paediatric septic shock management and were used in this study as the reference. Trainings done locally by Emergency triage assessment and treatment plus admission care (ETAT+) and Kenya Paediatric Protocols 2016 Guideline in Kenya do not focus specifically on septic shock but rather on signs of altered perfusion, which are applicable in septic shock recognition [18]. The current study aims at evaluating the prevalence and auditing the management practices at Kenyatta National Hospital (KNH) regarding septic shock in children. It is hoped that the study will provide the basis of development of local septic shock guidelines and tool kits for use in emergency care departments across the country.

## 2. Methods

A hospital based longitudinal study was carried out over a period of 2 months (September-October 2016) at the Kenyatta National Hospital after approval from the KNH/University of Nairobi ethics committee. KNH is a national teaching and referral hospital located in Kenya which provides emergency, outpatient and inpatient care. Being a referral hospital speciality care is also provided both as outpatient and inpatient. The study was carried out in paediatric emergency unit, paediatric wards, paediatric intensive care unit and new born unit. Children aged 0 days to 12 years are admitted in the paediatric section of the hospital and cared for by paediatric registrars (residents) and consultants. More than 90% of the children are referred from peripheral hospitals. Around 450 children are admitted in a month, where mortality of critically ill children is about 60%.

### 2.1. Objectives

The objectives of the study were to determine the prevalence, audit the management and determine the outcome at 72 hours of septic shock among admitted children.

### 2.2. Hypothesis

The management of septic shock in children at the Kenyatta National Hospital is as per the SSCG 2012.

### 2.3. Subjects

A study sample size of 325 was calculated using the Fischer's formula (95% confidence interval set at 1.96 and precision of 5%) with reference to study done by Basnet et al. in Nepal [19]. Children aged 0 days (term neonate ≥37 weeks) to 12 years admitted at the KNH paediatric wards, newborn unit (NBU) and PICU were included in the study. Children with birth asphyxia, trauma, burns, anaphylaxis, liver failure, known cardiac disease, chronic renal failure, diarrhoea and severe acute malnutrition were excluded. Consecutive sampling was done for data collection and all children were screened if they met the inclusion criteria. Informed consent was taken for all the enrolled children. The children received in the paediatric emergency unit and newborn unit are assessed by the paediatric registrars at any given time. The investigators closely assessed for signs of septic shock and children who were suspected to have septic shock were followed up for 72 hours.

### 2.4. Case Definition

A child was diagnosed to have septic shock when he/she had clinical signs of SIRS and all signs of abnormal perfusion (capillary refill time >2 s, cold extremities, weak or absent radial pulse and altered mental status), according to SSCG 2012 and ETAT+ developed by WHO [16, 18].

### 2.5. Data Collection and Analysis

A pretested questionnaire was used for data collection and recorded in the computer storage program MS-EXCEL at the end of 72 hours follow-up. The key measured documented variables audited were age, vital signs (temperature, respiratory and heart rate, and blood pressure), signs of altered perfusion (capillary refill time, temperature gradient, and radial pulse) and management (oxygen, blood sugar, fluids, antibiotics, urine output, calcium, lactate, blood, mechanical ventilation, and inotropes use). These are the maximum we could do due to limited resources with reference to SSCG 2012 guidelines. Hypotension was defined as <5^th^ centile for age as per the SSCG 2012 guidelines. The children diagnosed with septic shock only were followed up for 72 hours. Data was analysed using STATA 12 software comparing with the audit criteria. Audit data was compared to SSCG 2012 on paediatric septic shock management [1]. Frequency and percentages were calculated for categorical variables. Means with standard deviations were calculated for normal distribution and skewed data was expressed in terms of medians with interquartile ranges. Tests of association between the outcome variable (septic shock) and independent variables were carried out by chi-square test, *t*-test and logistic binary regression for normal distribution. Where distribution was skewed a Mann Whitney *U* tests was performed. Statistical significance was set at a *p*-value less than 0.05.

### 2.6. Ethics

A signed consent was obtained for all enrolled children. The study was approved by the KNH/UON ethics committee (P228/03/2016).

## 3. Results

**Figure 1:**
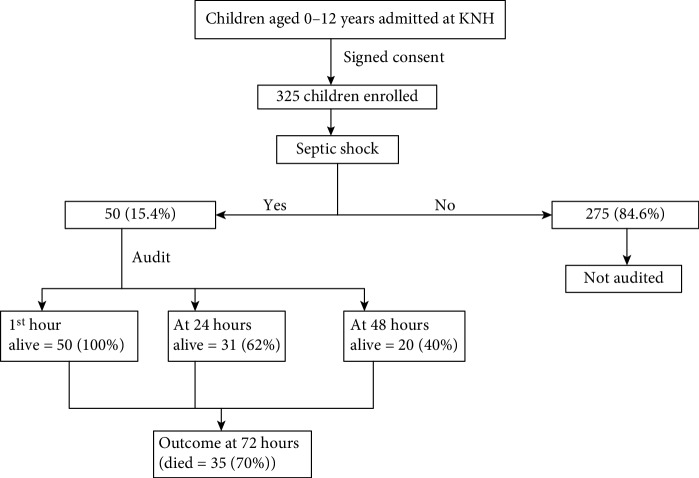
Study flow chart. 325 children were enrolled and 50 children were audited with septic shock.

A total of 325 children were analysed during the study period (Figure 1). Among these 58.8% were females. The median age was 8 months. Infants accounted for the majority of admissions but neonates had the highest proportion of septic shock (Table 1).

### 3.1. Prevalence of Septic Shock

Septic shock was diagnosed in 50 of the 325 children admitted, giving a prevalence of 15.4%. The median age was 4 months, neonates at 25.6% and infants at 20.9% formed the highest proportion of children with septic shock. None of the children above the age of >60 months was diagnosed with septic shock. Male: female ratio was 1:1.8. Low blood pressure was found by the investigators in 28 (56%) children. Young age (odds ratio 4.38 95% CI 1.37–8.24, *p* = 0.008) was significantly associated with septic shock.

### 3.2. Audit of the Management of Septic Shock

#### 3.2.1. Audit on Recognition of Septic Shock

Septic shock was recognized on admission by the attending health clinician in 28 (56%) of the children and the peripheral hospital by the attending health worker in 5 (11.4%) of 44 referred children with septic shock. The data on care of the referred children at the peripheral hospital was not available as most of the referral letters had incomplete documentation. The clinical signs that were not documented by the attending clinician but were important parameters for recognition of septic shock included capillary refill time in 14 (28%), radial pulse characteristics 13 (26%), temperature gradient 12 (24%) and altered consciousness 6 (12%). Oxygen saturation was measured in 40 (80%) and blood pressure was measured in only 10 (5%).

#### 3.2.2. Audit at the 1^st^ Hour of Recognition of Septic Shock

**Figure 2:**
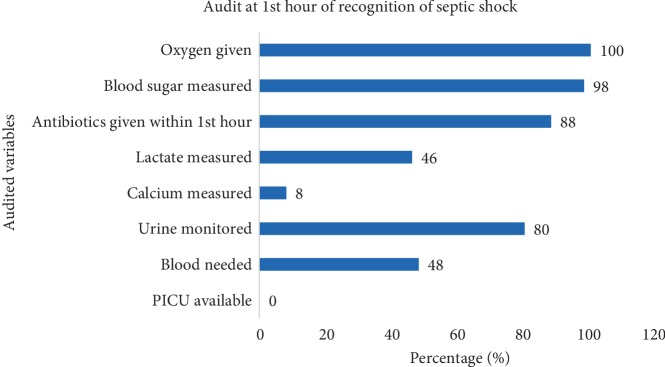
Audit of measured variables at 1^st^ hour of recognition of shock.

No child received optimum care and none received PICU/NICU care as per SSCG 2012 in the first golden hour of care as shown in (Figure 2). Sample for blood culture was taken in 12 (24%) children prior to antibiotic administration. Samples were not collected from 8 (21.1%) because the attending clinician did not order for sample culture and in 30 (79.0%) because of nonavailability of culture bottles.  Table 2 shows results and interventions audited for the first hour. Children who required inotropes and or airway management were admitted to PICU/NICU. The vasoactive agent was given according to the type of shock and children who needed respiratory support and had a GCS<8 were included for mechanical ventilation.

#### 3.2.3. Audit of Management of Septic Shock at 24 and 48 Hours

**Figure 3:**
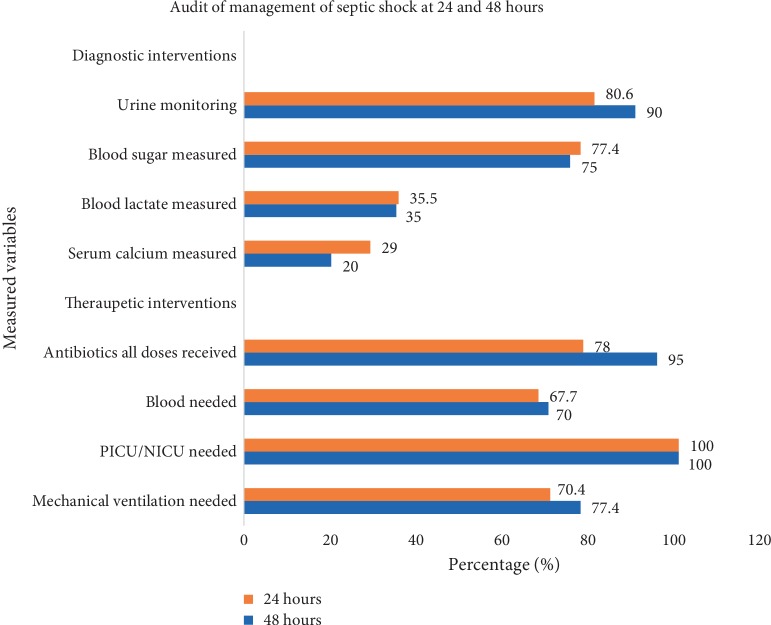
Audit of interventions measured at 24 and 48 hours of recognition of septic shock.

Among the children with septic shock 31 were alive at 24 hours and 20 were alive at 48 hours. Blood pressure was measured in only 19.4% and 20% at 24 and 48 hours respectively. The interventions audited at 24 and 48 hours are as shown in Figure 3. The results and interventions audited are shown in Table 3.

#### 3.2.4. Outcome of Septic Shock at 72 Hours

**Figure 4:**
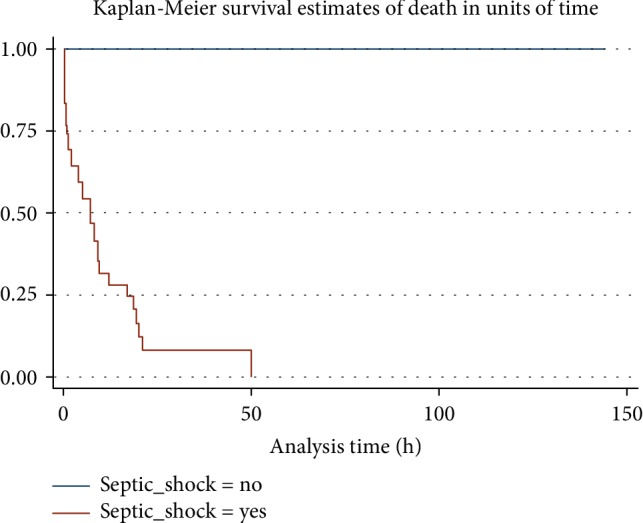
Kaplan–Meier survival curve showing estimate of death in hours in unit time.

At 72 hours follow up 35 (70.0%) had died with a median time of 14 hours. The largest number of children died within the 1^st^ 5 hours of admission and all deaths occurred within 50 hours of admission as shown in Figure 4. Infants had the highest proportion of mortality 19 (54.3%), followed by neonates 11 (31.3%) and 12–59 months old at 5 (14.3%). Out of the 35 children who died 22 (62.86%) were female, 29 (82.9%) were referrals and 6 (17.1%) were nonreferrals. The case fatality of septic shock at 72 hours was 55% in <1 month, 82.6% in 1–11 months and 71.4% in 12–59 months. Being a referral (odds ratio 2.89; 95%CI 1.16–7.20, *p* = 0.02)), unavailability of mechanical ventilator (odds ratio 5.33; 95%CI 1.08–26.36, *p* = 0.04) and having hypotension (odds ratio 10.0; 95% CI 2.31–43.16, *p* < 0.01) on admission were significantly associated with mortality at 72 hours. Age (<1 month (*p* = 0.3) or <12 months (*p* = 0.3)) and duration of follow up (≤24 hours, *p* = 0.71 and >24–≤48 hours, *p* = 0.8) were not significantly associated with mortality.

## 4. Discussion

The prevalence of paediatric septic shock among 325 children admitted at KNH was 15.4%, and it was higher than other similar studies done globally. A study done at a referral centre in India showed a prevalence of 2.2% of all admitted children [5]. Carvalho et al. working in Brazil reported prevalence of 9.8% for septic shock [20]. The prevalence of septic shock found in our study may have been higher than that reported from elsewhere partly because of the fact that three quarters of children studied were referrals from another hospital, with associated delay in recognition or transfer to KNH for better management.

The male : female ratio was 1 : 1.8 in our study while in a study done by Bindl et al. in 2003 it was 2 : 1 [21]. In our study we had more females diagnosed with septic shock but Bindl et al. showed Male gender has been associated with high mortality in sepsis and since most of our children were referrals from other public facilities, they may have died at the peripheral hospitals where shock may not have been recognized [21]. The study population was not systematically selected hence it is difficult to assess reason for high female proportion of septic shock.

In this study, a quarter of the neonates admitted had septic shock, being much higher than that found in other studies done globally. A study by Arizaga -Ballesteros et al. in two Mexican hospitals showed the prevalence of neonatal shock admitted to NICU to be 12.7% of all neonatal admissions [22]. The reasons for high neonatal prevalence of septic shock in this study were not explored but may be due to poor health seeking behaviour, negative traditional beliefs or poor maternal education on neonatal danger signs as seen from a study done locally by Michieka et al. (unpublished). Children aged less than a year comprised the highest number of admissions and diagnosis of septic shock. A study by Larsan et al. showed that infants had the highest prevalence of septic shock [23]. This can be explained by the low immune state that predisposes them to sepsis [24]. Our median age of 4 months is not very different to median age of 6 months reported by Larsen et al. [23]. In our study, no child above the age of 5 years was diagnosed with septic shock. This suggests that our main focus should be on children under 5 years of age for septic shock.

KNH has no specific guideline to diagnose or manage septic shock. It is thus not surprising that only 56% of the cases were recognized on admission. Paul et al. reported a higher recognition rate of 79% [25]. Lack of awareness may be the hindrance factor in recognition of septic shock in children; however, this aspect was not evaluated in this study.

The majority of our children were referrals. Hypotension is usually seen late in paediatric septic shock and this was found in 56% of septic shock children by the investigators. This suggest late diagnosis as well as delay in reaching KNH.

Management of septic shock appears to be problematic at KNH just like in many other public hospitals in developing countries. No child received optimum care over the first golden hour in our study with reference to SSCG 2012. The reasons behind inadequate management of septic shock were not fully evaluated in this study, but of note were factors like lack of knowledge on septic shock management, staff shortage, blood products unavailability when needed, inadequate laboratory support, unavailability of infusion pumps to give vasopressors in emergency departments, unavailability of monitors and PICU/NICU bed. Khilani et al. reported similar observations in a study in 2010 [26]. These findings are common in resource-limited countries globally and are a major limitation in management of children with septic shock.

Since this is the first study on paediatric septic shock in Kenya, our results cannot be compared to any other study locally, but studies done in other parts of the world show improvement in management of septic shock after educating health workers and implementation of guidelines. A similar study done in Utah highlights improvement in compliance after implementation of the guidelines [23]. Hence training of health care workers on septic shock remains of critical importance for improvement of management in this condition.

The initial 72 hours are critical in the management of septic shock and improvement in survival [13, 27]. In the continuation of septic shock care at 24 and 48 hours, clinical signs were recorded in a range of 19–100%. Blood pressure was measured only in less than a quarter of the patients and this may have been due to lack of proper cuff sizes in the wards. KNH has limited intensive care resources in terms of PICU/NICU bed availability, hence only few children manage to receive this care. We could not find a similar study to compare outcomes at 24 and 48 hours of audit of septic shock as most studies focus on the 1^st^ one-hour which is the golden hour in septic shock. Not all variables were measured as per SSCG 2012 Guidelines due to limitations on laboratory, equipment availability (monitors, blood pressure cuffs and staff shortage to closely monitor the children with septic shock).

The mortality at 72 hours from the time of recognition of septic shock in this study was 70%, while similar studies done elsewhere report mortality at 70% and 88.2% with an average of 4 days relating to delay in recognition of septic shock, lack of PICU infrastructure, understaffing and limited access to health care [15, 28]. The high mortality in our study may be due to unavailability of PICU/NICU beds during initial care, delay in recognition and early appropriate institution of management, and transfer from public health facilities to KNH and lack of awareness of the guidelines. Mortality was high in the initial 24 hours of admission of septic shock (54%). Children referred from other public hospitals and diagnosed with septic shock on admission at KNH were significantly associated with high mortality. This may be due to late referrals following the illness and unavailability of PICU/NICU care at KNH in the first hour of recognition of septic shock.

Mortality at 72 hours is not significantly predicted individually by age of the child with septic shock, sex and duration of stay in the hospital during this study. Similar results were seen in a study from India [29]. Infants had the highest case fatality in our study. Larsen et al. showed similar results though Cartaya et al. found lower mortality at 7% [23, 30]. Children diagnosed with septic shock and who needed ventilator care experienced significantly higher mortality. It is conceivable that improvement of critical care facilities would reduce mortality in such children.

The study has important implications for practice, local guidelines and further research regarding septic shock. The impact on practice can be through training to improve knowledge and skills in management of paediatric septic shock using SSCG 2012. The information obtained from this study will help improve on our resources. ETAT+ training does not go beyond basic care and hence training health care workers at KNH and lower level peripheral public hospitals on SSCG 2012 will improve recognition, reduce delay in transfer and care of septic shock. Giving feedback to the Ministry of Health and county governments will improve care in the lower level public hospitals.

The strength of this study is that it provides a basis for evaluating and improving the care of children with septic shock. The study was done in a public referral hospital with limited resources which reflect care in other peripheral public hospitals. The study transparently shows the reality of septic shock management in resource limited setting with no existing local guidelines and using SSCG 2012 as a reference.

The main limitations of this study were inadequate review of critical parameters due to limited resources such as laboratory, interventions and equipment availability in all paediatric units with reference to SSCG 2012. Lack of complete documentation from referral hospitals made it difficult to assess the care given prior to referring them to KNH. Barriers to the SSCG 2012 referred to earlier were not evaluated in this study.

## 5. Conclusion

The prevalence of septic shock was 15.4% with a mortality of 70% at 72 hours among 325 enrolled children. Septic shock was recognized by the attending clinician on admission in only 56% of the patients. There are no existing local guidelines on septic shock management and the standard of care provided with reference to Surviving sepsis Campaign Guidelines was not adequate. ETAT plus guidelines used in Kenya which are recognized by WHO do not focus specific on paediatric septic shock, hence there is need for training on SSCG and inclusion in to our Kenyan paediatric guidelines. Early recognition and management of septic shock requires continuous training of health care workers to create awareness and improve care.

## Figures and Tables

**Table 1:** Sociodemographic characteristics of enrolled children and those diagnosed with septic shock.

Variable	Characteristic	Enrolled children (*N* = 325)	Septic shock proportion
Age (months)	<1	78 (24.0)	20 (25.6)
	1–11	110 (33.9)	23 (20.9)
	12–59	96 (29.5)	7 (7.3)
	≥60	41 (12.6)	0 (0)
Sex	Female	191 (58.8)	36 (18.8)
	Male	134 (41.3)	32 (23.9)
Referred from another facility	No	91 (28.0)	44 (48.4)
	Yes	234 (72.0)	6 (2.56)

**Table 2:** Audit of interventions of septic shock during 1st hour after recognition of septic shock.

Variable	Results among those measured	At 1^st^ hour		
		Frequency (%)	Intervention	
			Done	*n*(%)
Blood sugar	<2.2 mmol/l	19 (38.9)	Corrected	18 (94.7)
	>10 mmol/l	20 (40.8)	–	–
Intravenous fluids 10–20 mls/kg/bolus	2 boluses (appropriate)	33 (66)	–	–
	0 bolus	1 (2)		
	1 bolus	12 (24)	–	–
	3 boluses	4 (8)	–	–
Antibiotics	Appropriate dose	49 (98)	–	–
	Mono therapy	32 (64)	–	–
	Dual therapy	17 (34)	–	–
	Triple therapy	1 (2)	–	–
Blood	Needed (Hb < 10 g/dl)	24 (48)	Available	3 (12.5)
Vasoactive agent	Needed	28 (56)	Available	0 (0)
Mechanical ventilation	Needed	42 (84.0)	Available	0 (0)

**Table 3:** Audit of the results and interventions at 24 and 48 hours.

Variable	Results among those measured		At 24 hours (alive = 31)			At 48 hours (alive = 20)		
			Frequency (%)	Intervention		Frequency (%)	Intervention	
				Done	*n*(%)		Done	*n*(%)
Urine output	<0.5 mls/kg/hr		10 (32.2)	Dialysis	0 (0)	2 (10.0)	Dialysis	1 (50)
	≥0.5 mls/kg/hr		15 (67.8)	–	–	16 (90.0)	–	
Antibiotic	Received all doses		25 (80.7)	–	–	19 (95.0)	–	
	Missed dose	1	4 (66.7)	IV access fixed	5 (83.3)	1 (100.0)	IV access fixed	1 (100)
		2	2 (33.3)			0		
	Antibiotics changed		7 (22.6)	–		1 (5.0)	–	
Blood	Needed		21 (67.7)	Available	2 (9.5)	14 (70.0)	Available	3 (21.4)
Blood sugar	<2.2 mmol/l		2 (16.7)	Corrected	2 (100)	0	–	–
	>10 mmol/l		10 (18.3)	Insulin	1 (10)	6 (30.0)	Insulin	1 (16.7)
PICU/NICU	Needed		31 (100.0)	Available	2 (6.5)	20 (100.0)	Available	4 (20.0)
Vasoactive agent	Needed		31 (100.0)	Dopamine	1 (3.2)	20 (100.0)	Dopamine	3 (15.0)
Mechanical ventilation	Needed		24 (70.0)	Available	2 (8.3)	14 (74.4)	Available	4 (28.6)

## Data Availability

The datasets used during the current study are available from the corresponding author on reasonable request.
